# Exploring Biological Predictive Factors of Progression After Surgery in High-Risk Renal Cell Carcinoma: Results From the French Cohort of the Randomized S-TRAC Trial Patients

**DOI:** 10.3389/fsurg.2020.00026

**Published:** 2020-06-05

**Authors:** Idir Ouzaid, Solène Florence Kammerer-Jacquet, Zineddine Khene, Alain Ravaud, Jean-Jacques Patard, Karim Bensalah, Nathalie Rioux-Leclercq

**Affiliations:** ^1^Department of Pathology, University of Rennes, CHU de Rennes, Rennes, France; ^2^Department of Urology, Bichat Claude Bernard Hospital, University of Paris, Paris, France; ^3^Department of Urology, University of Rennes, CHU de Rennes, Rennes, France; ^4^Department of Medical Oncology, CHU de Bordeaux, Bordeaux, France; ^5^Department of Urology, CH Mont de Marsan, Mont de Marsan, France

**Keywords:** angiogenesis, prognosis, progression, renal cell carcinoma, sunitinib, vascular endothelial growth factor

## Abstract

**Objective:** We aimed to explore biological predictive factors of progression after surgery in nonmetastatic renal cell carcinoma (RCC) using the collected tumors in the French cohort of the randomized S-TRAC trial patients.

**Patients and Methods:** We analyzed the tumors of the French cohort of STRAC that included 44 cases of clear cell RCC (ccRCC) that were collected from six centers. The main objective was to explore biological predictive factors of progression (defined as PFS) to sunitinib. Broad-spectrum analysis including immunohistochemistry, fluorescent *in situ* hybridization (FISH), comparative genomic hybridization (CGH) array, and transcriptomic analyses were performed on the tumors.

**Results:**Analysis of vascular density showed type 1 vascular stroma corresponding to high vascular density was associated with progression (*p* < 0.034). Loss of poly bromo-1 expression showed a distinct profile: a highly histopathological aggressive tumor with a marked angiogenic profile (vascular endothelial growth factor overexpression and immature vascular stroma type 2), no PD1 or PDL1 expression, and wild-type (WT) status of the *VHL* gene. There were 27 chromosome regions gained in patients with progression (on chromosomes 7 and 16, and to a lesser extent 8, 12, 17, 17, 19, 20 corresponding to 605 associated genes) and 10 regions lost in these same patients on chromosomes 8 and 9, and to a lesser extent 2 and 21 corresponding to 25 associated genes.

**Conclusion:**We found that an angiogenic phenotype defined by a high vascular density with a vascular type 2 stroma was a predictive factor of sunitinib resistance. Regardless of adjuvant treatment, chromosomal gains and losses and genomic alterations including *PBRM1* loss were associated with worse outcomes.

**Clinical Trial Registration**: ClinicalTrials.gov number, NCT00375674.

## Introduction

Renal cell carcinoma (RCC) is the most lethal urologic cancer, with an estimated 143,000 annual deaths (91,000 in men, 52,000 in women) (1). Surgical extirpation is the standard treatment of localized and locally advanced nonmetastatic disease (nmRCC) (2). However, up to 60% of patients with nonmetastatic locally advanced disease will experience recurrence at 5 years and a majority of these patients will die from the disease (3).

Tyrosine kinase inhibitors (TKIs) targeting vascular endothelial growth factor (VEGF) receptors have the ability to block the angiogenic pathway, and can impair tumor cell growth (4, 5). TKIs have been investigated in randomized controlled trials (RCTs) in the adjuvant setting to prevent progression in high-risk nmRCC after nephrectomy (6).

To date, the results of three of five of these trials have been reported (7–9). The S-TRAC trial was the only RCT to show a benefit of sunitinib in terms of progression-free survival (9). The two other trials (PROTECT and ASSURE) did not show any survival advantage in patients receiving adjuvant TKIs. These conflicting results might be related to the heterogeneity of study designs and patient selection, and there might be a potential benefit in a subgroup of high-risk nmRCC that has yet to be defined.

Our objective was to explore biological predictive factors of progression after surgery in nmRCC using the collected tumors in the French cohort of the randomized S-TRAC trial patients.

## Patients and Methods

### Patients

This ancillary study of the S-TRAC trial (ClinicalTrials.gov number, NCT00375674) included patients from six centers. Eligible patients were at least 18 years old and had a locoregional RCC (tumor stage III or higher, regional lymph-node metastasis, or both) on the basis of modified University of California Los Angeles Integrated Staging System (UISS) criteria (10). Other eligibility criteria included histologic confirmation of clear-cell RCC (ccRCC) and no previous systemic treatment.

### Treatment and Oncological Outcomes

After nephrectomy, patients were randomized in a 1:1 ratio to receive either sunitinib (50 mg per day) or placebo on a 4-weeks-on, 2-weeks-off schedule for 1 year. Dose interruptions or dose reductions to 37.5 mg per day were allowed, depending on the type and severity of toxicity. Treatment was pursued until disease recurrence, diagnosis of a secondary cancer, unacceptable toxic effects, or consent withdrawal.

The primary endpoint was disease-free survival, which was defined as the interval between randomization and first tumor recurrence, the occurrence of metastasis or a secondary cancer (as assessed by blinded independent central review), or death. Secondary endpoints included overall survival, safety, and health-related quality of life.

### Study Objectives

The main objective of this project was to explore biological predictive factors of progression (defined as PFS) to sunitinib.

### Immunohistochemistry

The immunohistochemical study was performed on 4-μm-thick sections of the addressed inclusion block. The main immunohistochemistry steps were dewaxing, rehydration, antigen detection, endogenous peroxidase neutralization, nonspecific site saturation, primary antibody, secondary antibody, amplification, revelation, and counterstaining. For the development of research antibodies, primary antibody concentrations, unmasking solutions, and amplification techniques were adapted to each antibody to define the most specific and sensitive labeling. Positive controls were obtained by referring to www.proteinatlas.org. Healthy tissues were chosen as controls over tumor tissues to avoid heterogeneity of immunological labeling on tumors. For the other antibodies, used routinely, the usual protocols were followed. Immunolabeling was then performed either manually (CXCR4) or on the Discovery XT® (Ventana, Tucson, AZ, USA) controller for the other antibodies for better reproducibility. The markers list and relevant interpretation are listed in Appendix 1. Vascular density was defined and evaluated as previously described (11).

### FISH Analysis

The interphase fluorescent *in situ* hybridization (FISH) technique was performed on 4-μm-thick sections. After dewaxing, an enzymatic treatment with pepsin from the sections was carried out, separating the DNA from the histones and thus facilitating the penetration of the probes. Once the double-stranded DNA was denatured, the probes could hybridize specifically to the region of interest. The ZytoLight® SPEC VHL/CEN 3 probe (Zytovision, Clinisciences, Nanterre, France) Dual Color Probe was used for VHL (locus deletion detection of the VHL gene to establish its status), the break-apart LSI MYC probe (Abbott, Rungis, France) for MYC (targeting the 8q region of interest), and the ZytoLight® SPEC MET/CEN 7 probe (Zytovision, Bremerhaven, Germany) for MET (completing the MET status).

The test probe was marked by the fluorochrome emitting in the green and by the control probe on the centromere marked by a fluorochrome emitting in the orange. The fluorescent signal generated by the probe when it was hybridized was visualized with an epifluorescence microscope. The nuclei were countercolored with di-amino-phenyl-indol (DAPI).

### CGH Array Analysis

The comparative genomic hybridization (CGH)-array technique was performed using tumor DNA from frozen tumor samples to assess differences in terms of either gains or losses of either whole chromosomes or subchromosomal regions. All samples were histologically checked for the presence of more than 50% tumor cells. Commercial DNA was chosen as the control DNA. After a DNA extraction (DNeasy® Blood & Tissue Qiagen® kit), the DNA was treated with RNase, then purified and quantified (spectrophotometer, Nanodrop®). After a digestion step by two restriction enzymes to obtain DNA fragments from 100 to 500 bp (SureTag DNA Labeling Kit, Agilent®), the tumor DNA and control DNA were labeled with two distinct fluorochromes (Cy5 and Cy3). They were co-hybridized on oligonucleotide sequences fixed on a solid support (4 × 180k chip, Agilent® with a resolution of 13 kb). The chips were read on a scanner (G2565CA, Agilent Technologies) that measures the fluorescence ratio for each locus. Data interpretation was performed using Cytogenomics software (version 2.0.6.0, Agilent Technologies), Hg19 database.

### Transcriptomic Analysis

Transcriptomic analyses were performed to evaluate the impact of genomic alterations. Total RNA was extracted from biological samples using AllPrep DNA/RNA Mini Kit (Qiagen, Hilden, Germany) according to the manufacturer's instructions with a maximum of 30 mg frozen tissue. Tissues were homogenized in RLT buffer using TissueLyser (Qiagen) followed by passing the lysate through a blunt 23-gauge needle. RNA isolation was performed according to the manufacturer's instructions. DNase digestion done also for RNA and integrity was checked using RNA 6000 NanoChips with the Agilent 2100 Bioanalyzer (Agilent, Diegem, Belgium). Only RNA preparations with an RNA integrity number (RIN) >6.9 were considered for further microarray analysis.

### Statistical Analysis

Differences between patients treated, respectively, with placebo and sunitinib were compared using the chi-square or Fisher tests for categorical variables (presented as proportions) and a nonparametric Wilcoxon rank sum test for continuous variables (presented as mean ± standard deviation, SD). Bonferroni correction was used for multiple comparisons. A univariate logistic regression model was constructed including pertinent variables. Significant variables on the univariate as well as significant differences on baseline characteristics were used to construct a multivariate analysis model to predict progression and progression under sunitinib. Statistical analysis was performed using R 3.0.0 (www.r-project.org) and *p*-values were two-sided, with statistical significance defined as *p* < 0.05.

## Results

### Patients and Treatment

The French cohort of STRAC included 44 cases of ccRCC that were collected from six centers. Overall, 40 patients met the study criteria were exploitable and frozen tissue was available from 25 patients. Baseline clinical and pathological findings were similar in both groups and are summarized in Table 1.

**Table 1:** Baseline patients' characteristics and pathological findings.

**Baseline patients' characteristics**	***n***	
Age at diagnosis (years, mean ± SD)	56, 6	± 4, 6 years
**Gender**		
Male	31	75 (%)
Female	10	25 (%)
High blood pressure	16	40 (%)
**Baseline performance status**		
ECOG 0	28	67 (%)
ECOG 1	13	33 (%)
Tumor size, mean ± SD (cm)	8,6	± 2.1 cm
**Nucleolar ISUP grade**		
Grade 2	6	16 (%)
Grade 3	23	61 (%)
Grade 4	9	24 (%)
Sarcomatoid component	5	13 (%)
**TNM staging**		
pT3a	10	26 (%)
pT3b	28	74 (%)
pN1	1	10 (%)
**Specific pathological features**		
Rhabdoïd component	1	3 (%)
Tumor necrosis	23	61 (%)
Vascular emboli	14	37 (%)
Perirenal fat invasion	19	50 (%)
Hilar fat invasion	21	55 (%)
Renal vein thrombus	10	26 (%)
Pyelocaliceal cavity invasion	3	8 (%)

Seventeen patients were included in the sunitinib arm (43%), of whom 10 patients had a dose reduction (25%). At the time of data collection, 11 (52.3%) and 10 (47.7%) patients had progressed in the placebo and sunitinib arms, respectively. Overall, eight had lung metastases, four lymph node invasion, and eight had various metastatic locations. Progression was similar in both arms.

### Immunohistochemical Findings

**Figure 1:**
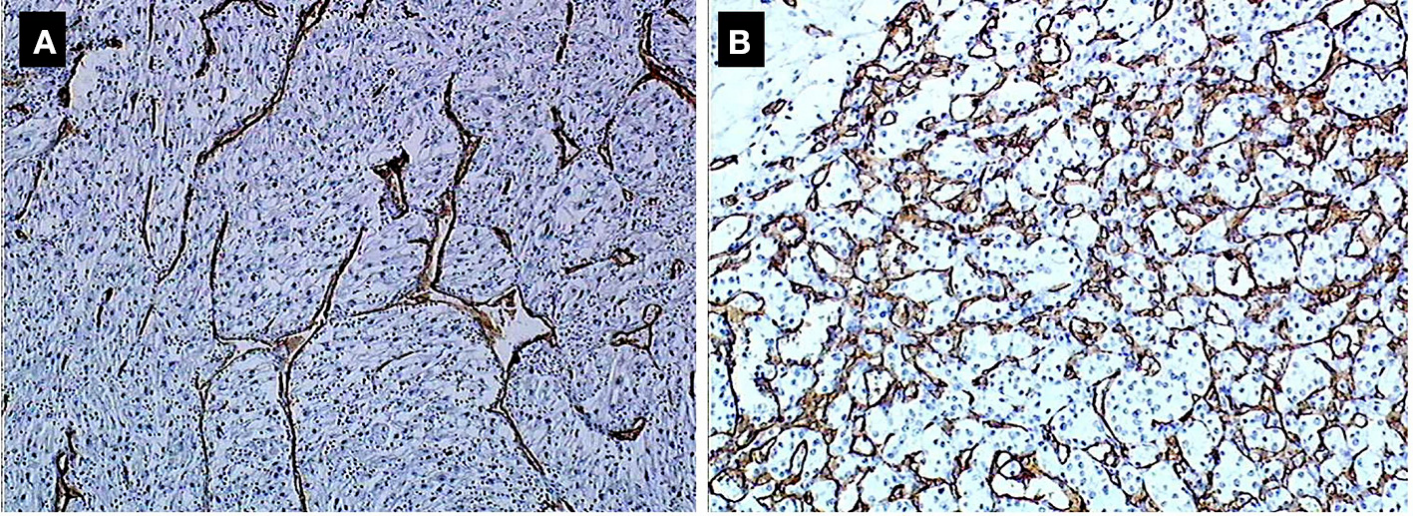
**(A)** Type 2 (low density). **(B)** Type 1 (high density) vascular stoma.

Immunohistochemically, 23 and 63.4% of tumors had an overexpression of CXCR4 and CAIX. Most tumors (65%) had a type 2 vascular stroma corresponding to a low vascular density and 35% had a mature type 1 stroma with a high vascular density (Figure 1). The mean expression of intratumoral VEGF was 36.8%. There was no significant difference for these biomarkers between the sunitinib group and the placebo group.

Tumors showed a higher peripheral lymphocyte infiltration (42%) than central infiltration (26%). There was no difference in immune phenotype as well as *VHL* status (deletion, mutation, or hypermethylation) in both sunitinib and placebo groups (Table 2).

**Table 2:** Immunohistochemical findings.

**Immunohistochemical findings**	***N***	
CXCR4 (%, mean ± SD)	23	±27
CAIX (%, mean ± SD)	63	±29
VEGF (%, mean ± SD)	36	±28
PD1 (2+3+)	7	(19%)
PDL1 (>1%)	5	(14%)
Mature stroma	13	(35%)
BAP1	37	(100%)
PBRM1	36	(97%)
TIL center (2+3+)	10	(26%)
TIL peripheral (2+3+)	16	(42%)
**VHL status**		
Mutation	25	(63%)
Deletion	5	(13%)

There was no loss of expression of *BAP1* or *PBRM1* except for one male patient who had a loss of expression of *PBRM1* reflecting an epigenetic mutation of *PBRM1*. This patient presented with a 10-cm tumor of International Society of Urologic Pathologists (ISUP) grade 3 without any sarcomatoid or rhabdoïd components. The tumor invaded the hilar and perirenal fatty tissue with a renal vein thrombus (pT3b) and necrotic alterations. Despite a massive lymphocyte infiltration in both the periphery and center of the tumor, there was no PD1 or PDL1 overexpression. VEGF was overexpressed in 100% of tumor cells with an immature type 2 stroma. The patient's *VHL* status was wild-type (WT). All these criteria are known to be poor prognostic factors (12). This patient developed lymph node and pulmonary metastases while he was included in the sunitinib arm (progression under treatment).

When comparing the two subpopulations of patients based on progression status, only the mature type 1 vascular stroma corresponding to high vascular density was associated with progression within the sunitinib arm (*p* < 0.034) (Table 3).

**Table 3:** Multivariate analysis of predictive factors of progression and progression after sunitinib.

	**Progression**	**Progression after sunitinib**
Tumor size (cm)	0.056	0.827
Fuhrman grade 2 vs. 3–4	0.378	0.375
Sarcomatoid features	0.355	1
Rhabdoïd features	0.447	1
Tumor necrosis	0.126	0.518
pT3a vs. 3b	1	1
pN	0.222	0.400
VHL mutation	0.567	1
VHL methylation	0.172	0.537
mCXCR4	0.668	0.826
nCXCR4	0.941	0.284
PD1	0.675	1
PDL1 (%)	0.393	0.473
VEGF (%)	0.933	0.169
CD31	0.173	0.034
BAP1	1	1
PBRM1	1	1
CAIX	0.778	0.686
TIL center	0.727	1
TIL peripheral	0.917	1

### CGH Array

**Figure 2:**
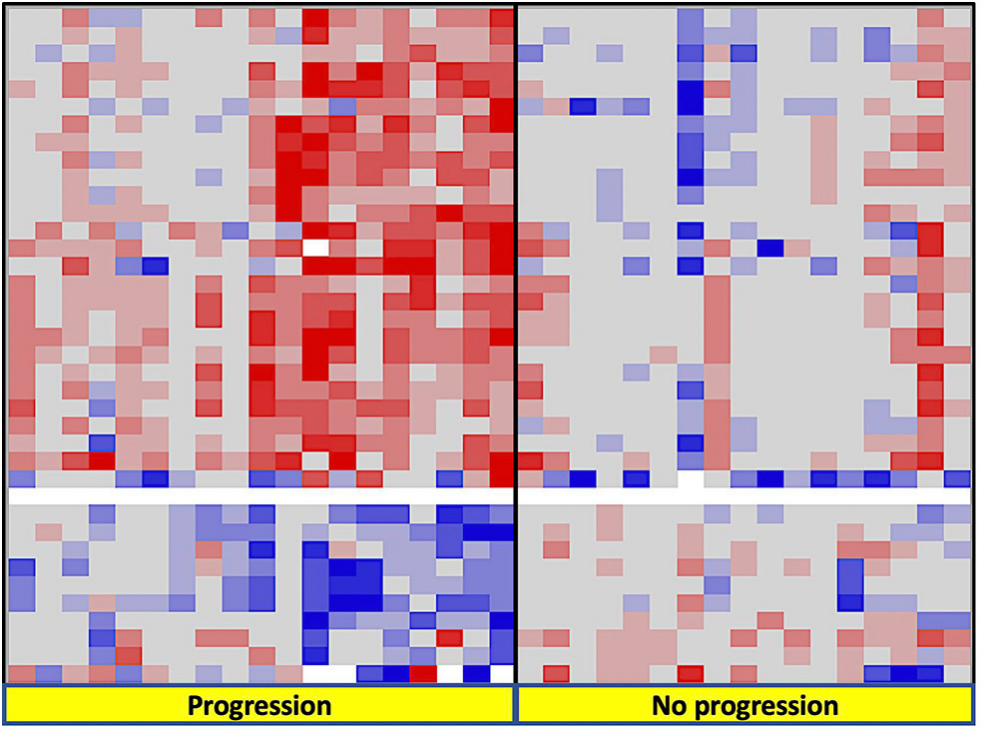
Findings on CGH arrays according to progression status during the follow-up after radical nephrectomy.

Subgroup comparison including patients (no treatment, no progression) vs. (no treatment progression) vs. (treatment, no progression) vs. (treatment, progression) no differential gained or lost chromosomal regions were noticed. However, comparing patients according to their progression status only, there were 27 chromosome regions gained in patients with progression (on chromosomes 7 and 16, and to a lesser extent 8, 12, 17, 17, 19, 20 corresponding to 605 associated genes) and 10 regions lost in these same patients on chromosomes 8 and 9, and to a lesser extent 2 and 21 corresponding to 25 associated genes (Figure 2).

### Transcriptomic and FISH Analyses

Transcriptomic and FISH findings did show any significant differences between both groups and their respective subgroups (TKI vs. placebo and progression vs. no progression).

## Discussion

Recent publications of three randomized trials aroused controversy regarding the utility of adjuvant TKI treatment after nephrectomy for high-risk RCC (13). S-TRAC was the only trial that showed a potential benefit in delaying progression in patients treated with sunitinib for 1 year after surgery (9). For this reason, we wanted to explore this specific cohort on a molecular perspective to provide some insights regarding predictive factors of progression in high-risk RCC patients.

We performed a large spectrum of analyses in the French cohort of the prospectively collected specimen of the S-TRAC trial including immunohistochemistry, FISH, and CGH array analyses.

Despite the limited sample size that could have impacted the results, three major findings emerged:

1. There was no histopathological phenotype, chromosomal, or transcriptomic difference between the sunitinib and placebo groups. In addition, patients in both arms showed similar progression rate in contrast with the whole population of the trial where there was a PFS benefit in the sunitinib arm. The absence of survival difference is mostly related to the small sample size, which accounts for 6.5% of the whole trial.
  Regardless of treatment arm, patients who progressed had more chromosomal gains and losses than those who did not. Recently, the TRACERx Renal Consortium published a comprehensive study picturing the genetic underpinnings and the evolutionary patterns of metastasis. Specifically, the hallmark genomic drivers of ccRCC metastasis are loss of 9p and 14q, which is in accordance with our findings (14).
  Selection of the best candidates for adjuvant treatment should probably take into account molecular patterns of the tumors besides macroscopic and pathological findings after nephrectomy. Different subtypes of primary tumors harbor different courses after radical treatment. For example, primary tumors with low intratumor heterogeneity (ITH) and a low fraction of the tumor genome affected by somatic copy-number alterations (SCNAs) have a low metastatic potential (15). Primary tumors with high ITH are associated with a low risk of progression, whereas primary tumors with low ITH but elevated SCNAs are associated with rapid and multilocular dissemination (15).
2. The only patient in the cohort with loss of *PBRM1* (poly bromo-1) expression showed a distinct profile: a highly histopathological aggressive tumor with a marked angiogenic profile (VEGF overexpression and immature vascular stroma type 2), no PD1 or PDL1 expression, and WT status of the *VHL* gene. Despite the randomization in the sunitinib arm, the patient developed early metastases.
  Loss of *PBRM1* requires special attention because it is the most frequently mutated gene after *VHL* (16). *PBRM1*(chromosome 3p21) encodes the BAF180 protein, which is a subunit of the ATP-dependent chromatin remodeling complex called SWI/SNF (SWItch/Sucrose Non-Fermentable). In ccRCC, most *PBRM1* mutations lead to the loss of the protein (17). As shown here, clinical data suggest that negative expression of *PBRM1* is correlated with advanced tumor stage, low differentiation grade, and worse patient outcome (18, 19). In addition, *PBRM1* patients express neither PD1 nor PDL1. Therefore, they might also be resistant to checkpoint inhibitors.
3. The only significant predictive factor for progression in patients treated with sunitinib, was the presence of a subtype 1 vascular stroma corresponding to a high vascular density. We are aware of the limited sample size of our cohort but we think this finding can raise hypothesis. Microvessel density (MVD), which is a surrogate of angiogenesis, has been suggested to predict prognosis of patients with RCC, but its ability to predict survival of patients with RCC remains controversial (20). A recent meta-analysis suggested MVD was not reliably associated with survival, which may reflect the need to consider whether the microvasculature is differentiated or not. A study showed two distinct types of microvessels can be identified in ccRCC: undifferentiated (CD31+/CD34–) and differentiated (CD34+) vessels. Differentiated MVD was significantly correlated with lower tumor grade and longer survival (21). Vessel density measurement can predict response to therapy with high sensitivity and specificity (22). The exact significance of MVD remains to be investigated in larger cohorts.

Our exploratory and hypotheses generating study aims to biologically identify, among all patients with high-risk localized RCC based on the UISS grading system, who might derive more benefit from adjuvant sunitinib. Accordingly, a recent study assessed the correlations between 11 single-nucleotide polymorphisms (SNPs, including specific SNPs in VEGFA, VEGFR1, and VEGFR3) and disease-free survival (DFS) and overall survival (OS) were assessed in another sub-subset of S-TRAC trial patients. Three of the 11 SNPs demonstrated improved DFS with sunitinib treatment over placebo with hazard ratios (HRs) between 0.44 and 0.56, compared to 0.76 for the overall population. This improvement in HR supports a predictive value for this biomarker defined subset of patients receiving sunitinib treatment. In particular, the genotypes C/C for VEGFR1 rs9554320, T/T for VEGFR2 rs2071559, and T/T for eNOS rs2070744 were associated with a longer DFS with sunitinib vs. placebo treatment (23).

Selecting patients with biological features of worst outcomes could be combined with recurrence assessment tools such as the 16-gene signature recurrence score (24). This score has been validated on the S-TRAC trial subgroup population and showed significance in both arms, with the strongest results in the placebo arms (25).

The major limitation of our study was sample size. In fact, the study could not be conducted on the whole S-TRAC population but only on patients accrued in French centers. This limited sample size had an impact on the results by decreasing the strength of statistical modeling. In addition, comparison within each group (treatment arm vs. placebo arm) were limited. However, despite the sample size, performing a comprehensive analysis with various molecular analyses allowed a thorough exploitation of the prospectively acquired clinical data and biological samples. We should acknowledge this can only be hypothesis generating to show directions of future research in the field of patient treatment for an nmRCC with high risk of progression.

Finally, our findings could be extrapolated to the metastatic setting for treatment selection. TKIs, now combined with immunotherapy (anti-PD1), have been shown to be effective as first-line treatment of metastatic RCC in recently published phase III trials (26–28). This suggests a remaining role of angiogenesis targeting therapies in the immunotherapy era. Updated EAU guidelines recommend the use of either immunooncology (IO)–IO (ipilimumab and nivolumab) or IO–TKI (pembrolizumab and axitinib or avelumab and axitinib) combinations in the first-line treatment of mRCC (29). These options were all reported to show a survival benefit compared to conventional sunitinib alone. However, there is no clinical direct comparison between the two combination strategies. Therefore, a treatment plan could rely on tumor biological profiling based on biopsy or nephrectomy specimen analyses. In fact, an immunologic tumor type (high PDL1 expression, CD8 T-cell infiltration, sarcomatoid features) is thought to benefit more from an IO–IO combination while IO–TKI would be more suited to an angiogenic type (high vascular density) (30, 31). As shown here, vascular phenotype (type 1 vs. type 2) using MVD analysis could help treatment selection of one or the other approach (IO–IO vs. IO–TKI).

## Conclusion

In a population of patients who had nephrectomy for a high-risk RCC, we found that an angiogenic phenotype defined by a high vascular density with a vascular type 2 stroma was a predictive factor of sunitinib resistance. Regardless of adjuvant treatment, chromosomal gains and losses and genomic alterations including *PBRM1* loss were associated with worse outcomes. These findings reinforce the role of tumor biology in treatment selection in the management of RCC.

## Data Availability Statement

The raw data supporting the conclusions of this article will be made available by the authors, without undue reservation, to any qualified researcher.

## Ethics Statement

The studies involving human participants were reviewed and approved by CHU de Rennes. The patients/participants provided their written informed consent to participate in this study.

## Author Contributions

IO: drafting of the manuscript and data analysis. SK-J: data acquisition and drafting of the manuscript. ZK: data acquisition, statistical analysis, and editing. AR and J-JP: study design, editing, review of the manuscript. KB and NR-L: study supervision and revision of the manuscript.

## Conflict of Interest

AR: reports receiving fees for serving on advisory boards from Pfizer, Novartis, GlaxoSmithKline, Bristol-Myers Squibb, and Roche; lecture fees from Pfizer, Novartis, and GlaxoSmithKline; travel support from Pfizer, Novartis, GlaxoSmithKline, Bristol-Myers Squibb, and Merck Sharp & Dohme; and grant support from Pfizer and Novartis. KB: research grant and consultancy for Pfizer, Intuitive Surgical. The remaining authors declare that the research was conducted in the absence of any commercial or financial relationships that could be construed as a potential conflict of interest.
