# Supplementary material for: Exploring Biological Predictive Factors of Progression After Surgery in High-Risk Renal Cell Carcinoma: Results From the French Cohort of the Randomized S-TRAC Trial Patients
Source: Front Surg. 2020 Jun 5;7:26. doi: 10.3389/fsurg.2020.00026 (PMC7289956; doi:10.3389/fsurg.2020.00026)
Supplement: Supplementary file 1 [file Table_1.DOCX]

**Appendix 1: Immunochemistry analyses**

**Used antibodies**

**CAIX:** EDTA pH 8, polyclonal rabbit antibody, ab15086, Abcam® 1/1500-60mn Kit DAB Map

**PD-1:** EDTA pH 8, monoclonal mouse antibody, ab 52587, Abcam® 1/400-32mn Kit Omni Map

**PD-L1 R&D:** EDTA pH 8 Ac monoclonal mouse clone 130021, R&D systems 1/400-60mn Kit Omni Map

**PD-L1 sp142:** EDTA pH 8, monoclonal rabbit antibody, sp142, Optiview kit®, Spring Bioscience®.

**VEGFA:** EDTA pH 8, monoclonal rabbit antibody sp28, DAB Map Kit®, Spring Bioscience®

**CD31:** Monoclonal antibody, clone JC70A, Dako®

**BAP1:** sc-28383 antibody BAP1 (C-4), Santa Cruz Biotechnology®

**SETD2:** SETD2 antibody, Clone HPA042451, Sigma-Aldrich®

**PBRM1:** A301-591A rabbit anti-PB1/BAF180, Euromedex®

**CXCR4:** Polyclonal rabbit antibody, ab2074, Abcam®

**Immunochemistry marker interpretation**

**CAIX:** Cytoplasmic or membrane tumor positivity % of cells low to intense (0-3) >85% (Patard JJ, Int J Cancer 2008)

**MET:** Cytoplasmic or membrane tumor positivity % of cells low to intense (0-3) (Shin SJ, Oncologist 2015)

**PAR3:** Cytoplasmic or membrane tumors % of cells low to intense (0-3) (Dugay F, Int J Cancer. 2014)

**PD-1:** Cytoplasmic immune absent to dense (0-3) low to intense (0-3) (Thompson RH, Clin Cancer Res 2007)

**PD-L1 R&D** cytoplasmic or membrane tumor positivity % of cells low to intense (0-3) Expression >5% Intensity 2-3 (Thompson RH Cancer Res 2006, Shin, Shin SJ, Oncologist 2015)

**PD-L1 sp142:** Cytoplasmic or membrane tumor positivity % of cells low to intense (0-3) Expression >5% Intensity 2-3 (Thompson RH Cancer Res 2006, Shin, Shin SJ, Oncologist 2015)

**VEGFA:** Cytoplasmic or membrane tumor positivity % of cells low to intense (0-3) >30% (Patard JJ, Br j Cancer 2009).

**CXCR4:** Marking was membrane or cytoplasmic, sometimes nuclear. The percentage of marked tumor cells was reported (Wang L, World J Surg Oncol, 2012)

**CD31:** The labelling of endothelial cells was membranous. It allowed assessment of microscopic vascular density (MVD) and vascular phenotype. To determine the MVD, the 3 highest density areas were defined and the number of vessels was counted per mm^2^. The MVD was obtained by averaging these 3 accounts. The angiogenic phenotype was considered mature, noted 1, when it was like the vascularization of the normal kidney. It shows small, round, numerous and frequently anastomosed vessels within the tumor. These vessels maintained normal development and form few branches. The angiogenic phenotype was immature, noted 2, when it differed significantly from the vascularization of the normal kidney. The vessels were elongated, stretched and large. They were rarer in the tumor. It showed an aberrant development with many ramifications

**BAP1, SETD2 and PBRM1:** loss of expression or not of tumor cells >1%.
